# Performance of a single slope solar still using different porous absorbing materials: an experimental approach

**DOI:** 10.1007/s11356-023-27465-5

**Published:** 2023-05-12

**Authors:** Khaled Ramzy, Mohamed Abdelgaleel, Abd Elnaby Kabeel, Heba Mosalam

**Affiliations:** 1 Department of Mechanical Engineering, Suez Canal University, Ismailia, Egypt; 2 Faculty of Technology and Education, Suez University, Suez, Egypt; 3 Mechanical Power Engineering Department, Faculty of Engineering, Tanta University, Tanta, Egypt; 4 Electro-Mechanics Department, Heliopolis University for Sustainable Development, Cairo, Egypt; 5 Faculty of engineering, Delta University for Science and Technology, Gamasa, Egypt

**Keywords:** Performance, Productivity, Solar still, Desalination, Efficiency, Exergo-economic

## Abstract

Desalination is a critical process to address water scarcity in arid regions worldwide, and solar stills provide an economical solution despite their productivity limitations. This study aimed to enhance the performance and productivity of solar stills by constructing two stills with different natural and artificial absorbing materials such as black luffa, luffa, fine steel wool, and steel wool pads. The solar stills were tested in Egypt under comparable weather conditions, and their productivity, solar intensity, wind velocity, and temperature were measured to determine their thermal efficiency and exergo-economic analysis. Results showed that the choice of absorbing material significantly impacted solar still productivity, with steel wool pads achieving the highest yield of 4.384 l/m^2^. Moreover, steel wool pads also exhibited the highest thermal efficiency at 32.74%. The cost per liter (CPL) was the lowest with steel wool pads at 0.0034 $/l/m^2^. Finally, the payback period and exergo-economic analysis demonstrated that incorporating steel wool pads was the most promising modification for enhancing solar still performance compared to other modifications.

## Introduction

The provision of potable water has always been a major concern, dating back to the days of travel and war. However, with the onset of climate change and the drying up of many rivers, it has become increasingly essential to have multiple sources of potable water. As a result, the importance of water has been included in the sustainable development goals, which many countries strive to implement to ensure their citizens have access to basic necessities. One of the easiest and most straightforward ways to obtain potable water is through solar water desalination. While it was previously used without any incentives to increase water production or improve drinking water quality, numerous studies have since been conducted using different techniques and types of basins or materials for seawater filtration. Many early studies have explored the use of solar concentration methods to increase renewable energy usage. To improve system productivity and efficiency, researchers have utilized different types of solar power, such as storing sensible heat using a metal matrix structure, as described in Dhandapani et al. (2019), which enables faster system start-up and reduces heat losses within the basin. Other researchers have experimented with using gravel as a storage material, combined with tracking parabolic trough and tubular solar still, as detailed in Elashmawy (2020), Results have shown that using gravel enhances energy efficiency by 13% and productivity yield by 14%. Additionally, the use of PTC increases productivity by almost nine times and reduces the cost of liter production by about 12%.

According to research, using inclined and tubular solar technology with single tracking parabolic trough heating can increase productivity to 35.62% and produce 7.8 l of fresh water per day, while significantly decreasing production costs (Ahmed et al. 2022). In terms of water depth, a study using a simple single-slope solar still system combined with a parabolic trough found that a 5 cm water depth was optimal for freshwater productivity, energy efficiency, and earned carbon credit mitigation (Kumar et al. 2020). Another study supported this finding, showing that productivity increased with shallower water depths in the basin (Bhargva and Yadav 2021). In a separate study, modeling a single slope solar still with different basin water depths and phase change materials as energy storage, magnesium sulfate heptahydrate was found to be the most efficient in water distillation (Somanchi et al. 2015).

In terms of capillary rise and material porosity, research has shown that using wick material with wire mesh such as water coral fleece with a weir mesh stepped absorber plate produces better performance in solar stills (Hansen et al. 2015). Additionally, using wick with multiple v-shaped floating single-slope solar still has been tested, resulting in a 26% increase in surface area and a 20% increase in fresh water productivity with the conservation solar still (CSS) (Agrawal and Rana 2019).

Several studies have explored different methods to enhance freshwater production in solar still systems. One such method involves using wick materials as porous absorbers in single and double pyramid slope-shaped solar stills to investigate heat transfer and Nusselt number (Wu et al. 2017). Dried pond fibers have also been used in the basin to increase freshwater productivity, with the results showing that using five dried pond fibers increased productivity and decreased payback period and cost per freshwater production. Another study compared the payback period, energy, exergy, and productivity of using graphite plat fines and magnet (GPF-MSS) with traditional methods (Dhivagar and Mohanraj 2021). The results showed an increase in productivity by 19.8%, energy with 21.4%, and exergy efficiency with 18.1%, and the production met the Bureau of Indian Standard (BIS) requirements. A literature review of solar stills and the techniques used to enhance freshwater production, such as glass cover, absorber plate, inlet water temperature, glass angle, and water depth, was presented in Zala et al. (2013). Additionally, a literature study for the main parameters and designs of light-to-heat systems in solar stills was presented in Chamsa-ard et al. (2020). Six solar distillation systems, including conventional solar still (CSS), CSS combined with a parabolic trough collector, CSS with steel wire mesh in the basin, CSS with wire mesh and PTC, CSS with sand in the basin, and CSS with sand and PTC, were studied in different weather conditions (Hassan et al. 2020). The findings suggest that using sand inside the basin with a parabolic trough collector results in the maximum freshwater yield during the summer, with a 1.21% increase compared to CSS and 102.1% increase compared to CSS, SD, and PTC in winter.

Various systems have been explored to improve the efficiency of solar still units, with the parabolic trough system being one of the most effective methods of speeding up the evaporation process (Mosalam and Hassan 2020). Miniature and industrial-sized parabolic systems have been developed, with some systems generating both electrical and thermal energy and producing up to 4 l of water per minute at temperatures of up to 72 °C. Researchers have also investigated the use of Fresnel lens FLR to enhance solar concentration in single-slope solar stills, demonstrating effectiveness for larger water depths (Johnson et al. 2019).

In the context of seawater evaporation and condensation enhancement using single-slope solar still units, researchers have explored the use of natural materials such as molasses, rice husk, sawdust, bamboo straw, and banana leaf powder (Natarajan et al. 2022). The use of sawdust and rice straw resulted in a 62.88% improvement in output per square meter compared to traditional solar stills (Natarajan et al. 2022). Luffa acutangula fibers have also been employed to enhance productivity by 25.23% compared to conventional solar stills (Suraparaju and Natarajan 2021). However, the use of ridge gourd fibers was found to be insignificant in enhancing the efficiency of single-slope solar still systems (Suraparaju and Natarajan 2020).

Another study explored the use of various fibers and materials, including floating coal, cotton fabrics, and nanoscale carbon black particles, to enhance thermal performance (Sharshir et al. 2021). Three scenarios were investigated, with modified solar still-C (carbon black nanoparticles dispersed on top of coal/cotton combination) producing the largest increases in cumulative yield, average energy efficiency, and average energy efficiency when compared to the reference solar still, at 59.33%, 75.12%, and 142.7%, respectively. This approach may also help reduce production costs by 25.32% and boost carbon emission reductions by 127.5% (Sharshir et al. 2021).

To enhance the efficiency of a single-slope solar still system, another approach is to use phase change material (PCM) as a numerical model with varying melting temperatures. This allows excess solar energy to be stored in the morning and used later at night. The choice of PCM is affected by the maximum temperature that can be reached by the brackish water basin, as reported in Ansari et al. (2013). Several PCM materials have been applied to solar still systems, including potassium dichromate (K_2_Cr_2_O_7_), magnesium sulfate heptahydrate (MgSO_4_ 7H_2_O), and sodium acetate (CH_3_COONa), with the latter two showing better productivity of potable water (Gugulothu et al. 2015). In reference (El-Sebaii et al. 2009), using 3.3 cm of stearic acid as a PCM increased productivity from 4.99 kg/m^2^ day to 9.005 kg/m^2^ per day in the summer. It is recommended to use stearic acid with wick mesh techniques to enhance productivity at night in single-slope solar still systems.

In another study comparing two solar still systems, sensible heat was advanced to improve evaporations. Two methods were employed in Thakur et al. (2021): one experiment used reduced graphene oxide (SS-RGO), and the other added an active carbon pellet (SS-RGO-ACP). The results showed that using SS-RGO-ACP had better energy and thermal performance, achieving 1.04 t/year based on energy goal CO_2_ mitigation.

Lastly, cooking oil was reused in the basin to increase sensible heat and hence increase evaporation, resulting in an increase in freshwater productivity from 3.02 to 3.77 l per meter square. The energy efficiency was 24.35% more, and the exergy was 0.69% (Balachandran et al. 2020).

According to a study conducted in Saudi Arabia, a tubular solar still device was developed with composite sensible heat storage tubes (CSHSTs) containing silica-filled sand and copper wire in the middle. This device was combined with a parabolic concentrator solar tracking system consisting of 12 tubes and used with saline water. The results showed a 24.05% enhancement in freshwater yield and a 20.06% enhancement in thermal daily efficiency with lower production costs than traditional solar still devices (Elashmawy and Ahmed 2021).

A double-effect passive solar still was designed to determine the amount of freshwater collected by the solar still. Using CATIA and ANSYS FLUENT for the transient state, CFD simulation was used to simulate the production rate, which was found to be in agreement with the experimental results (Nadgire et al. 2020). ANSYS FLUENT was also used in 2021 to validate the simulation and experiment of using coarse aggregate in solar still (Dhivagar et al. 2021b).

The temperature distribution along the single-slop solar system was studied and analyzed by the explicit finite difference method, which found that the temperature gradient from the basin to the glass cover was about 65 °C (Yeo et al. 2014). The correlation model by Dunkle’s heat transfer relations was used to predict the thermal performance by using sponge liner (Sengottain et al. 2014).

Experimental research was conducted to investigate the evaporation and condensation processes of brackish water that had been distilled utilizing a direct solar distiller and chemical solution. It was concluded that the amount of distillate produced substantially doubles when a chemical color is used, with thymol blues producing slightly more than orange methyl. The productivity of the insulated distiller was also found to be much higher than that of non-insulated distillers (Abed et al. 2022). A copper condenser was also used to enhance the productivity of the CSS by about 7.5% with a cost-effectiveness of 55% compared to the CSS (Nehar et al. 2022) Additionally, a crushed gravel sand and biomass evaporator assisted solar still was used with an exergy efficiency 35% higher than the CSS (R Dhivagar et al. 2022a).

In a recent study, natural and artificial porous absorbing materials such as luffa, black luffa, fine steel wool, and steel wool pads were used to enhance the productivity of the solar still. These materials led to an increase in the evaporation rate by enhancing the water surface area and improving the solar still’s performance. Different experiments were performed under the same climate conditions, and the solar still’s performance was enhanced and compared with the aid of economic evaluation for each one. Furthermore, exergoeconomic analysis was evaluated based on the cost and productivity of the solar still using natural and artificial porous absorbing materials.

## Experimental setup and procedure

**Fig. 1:**
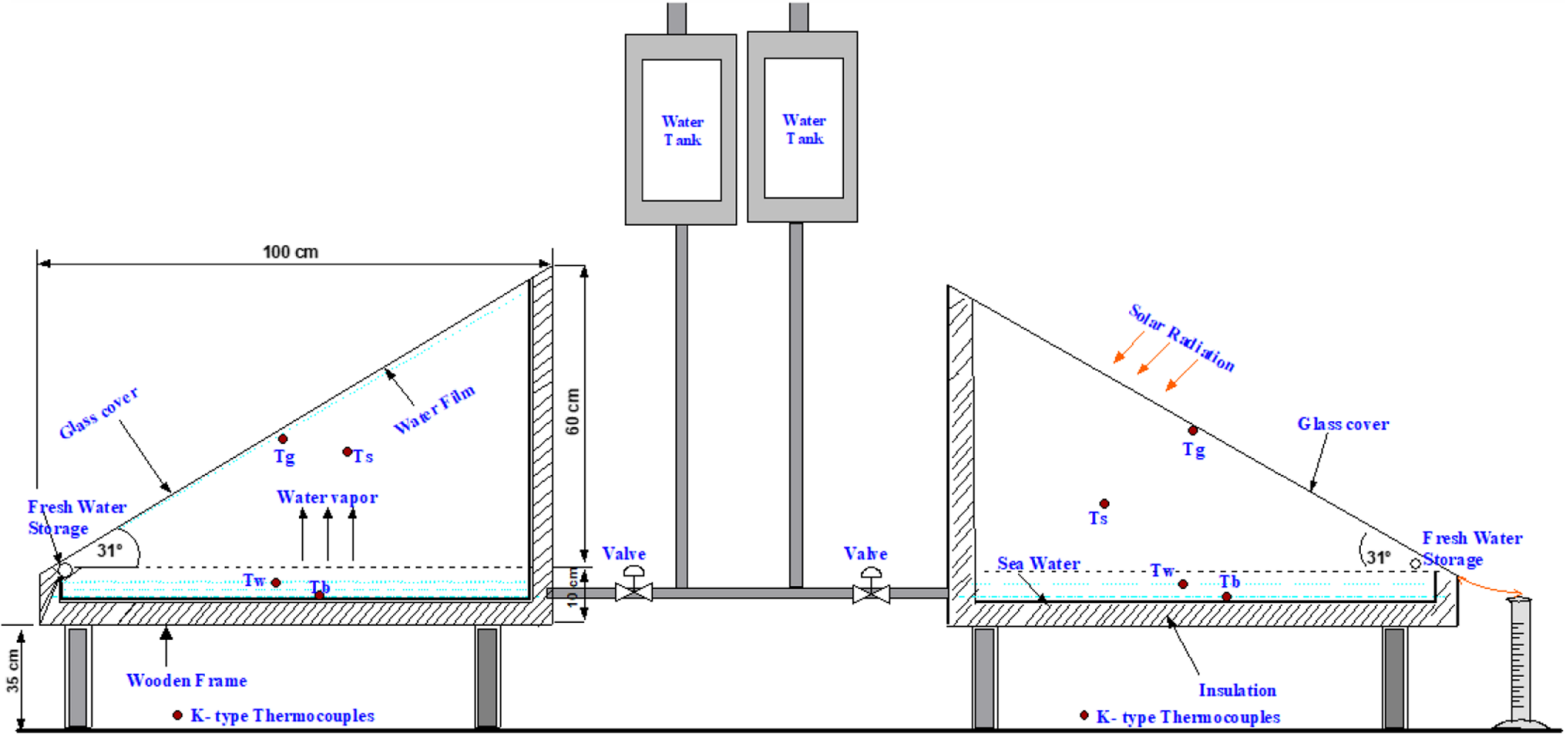
Schematic drawing for the experimental setup

**Fig. 2:**
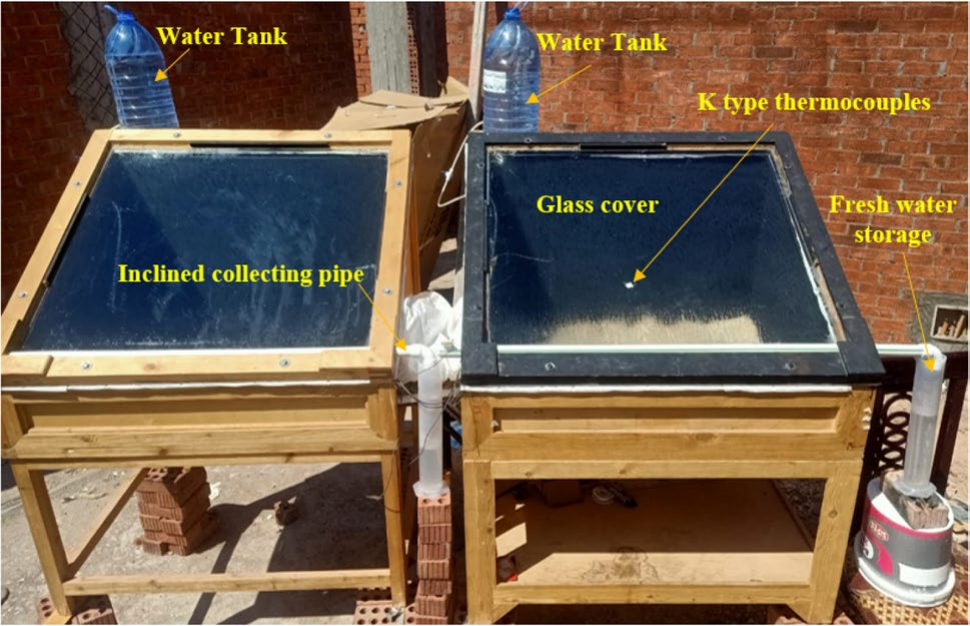
Photograph of the experimental setup

In this section, the experimental setup and procedure are described, including the measured parameters and measuring tools. The setup was created, tested, and put into place at the solar energy laboratory of Suez Canal University’s Faculty of Engineering in Egypt. Two solar stills were used in the experiment, one as a conventional mode and the other as a modified solar still with a water tank and piping connections. The design and schematic drawing of the experimental setup are displayed in Fig. 1. K type thermocouples were used to measure various temperatures, including the absorber, water, environment, and glass. The surrounding temperature, solar irradiation intensity, and wind speed were also measured. The complete setup is shown in Fig. 2. The output from the stills was collected in a trough and drained to an external bottle. The solar stills were mounted on a frame and well-insulated, and the basin was 0.8 m^2^ with a 10 cm low-side wall and a 70 cm high-side wall. The stills were painted black to increase solar absorptivity, and foam insulation measuring 5 cm thick was used to insulate the system’s side walls and basin. The still was sealed with silicone rubber sealant to prevent leaks. The glass cover was positioned at a 31° angle on the edge of the circular side.

### The conventional solar still with luffa fibers

Luffa fibers were utilized in the tested solar still basin to increase the floating water surface area with heated air inside the still. Additionally, the luffa fibers lessen the surface tension between the water molecules, which causes the water molecules to evaporate quickly. The molecules quickly and easily depart the free water surface, headed for the glass cover. Luffa fibers were utilized in this experiment in a normal distribution inside the still in each trial, with a 3 cm water depth. In a different experiment, the luffa fibers were painted black to boost solar energy absorption.

**Fig. 3:**
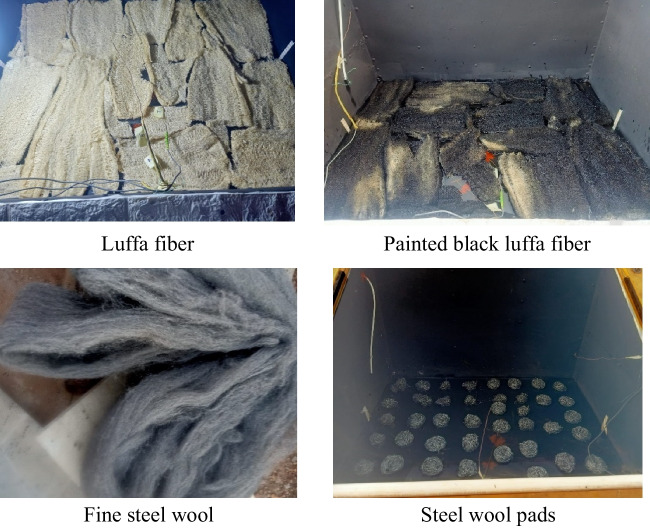
Photograph of luffa fiber, painted black luffa fiber, fine steel wool, and steel wool pads in the experimental setup

All experiments were done for several days to compare the outcomes on closed days and select the results that have approximately the same weather characteristics. A photograph of the luffa fibers, painted black luffa fibers, fine steel wool, and steel wool pads is shown in Fig. 3.

## Instruments and measurements

In this section, the equipment used to measure various parameters including ambient temperature, total dissolved solids, solar radiation, and temperatures inside solar stills is described. To determine the total solar radiation on a horizontal surface during the experimental days, a solar power analyzer was used. The analyzer measures the total amount of solar radiation coming from all angles and is fixed to a table that is moved horizontally using adjustable leveling screws and a built-in water balance. The sensor output data is recorded on a digital output screen and is calibrated to measure the amount of solar radiation in watts per square meter.

To measure the temperatures inside the solar still, eight k-type thermocouples were fixed at different positions. One sensor was used to measure the basin surface temperature (T_b_), another was used to measure the water temperature (T_w_) in the middle of the basin, and one each was used to measure the air temperatures inside the solar still and the glass cover (T_g_). The thermocouples have a resolution of 0.1 °C and accuracy (0.3% rdg + 1 °C) and are programmable with four channels and one button press to show T_1_-T_2_ and an indication about the range with error messages.

The fresh and salty feed water TDS were measured at the start and conclusion of the workday using an HI9813-61 portable meter, which automatically switches between various conductivity and TDS units and is easy to use without the need for troubleshooting. In addition, the output water productivity was measured using a graduated jar cylinder. Wind velocity is an important factor that impacts the performance of the solar still, and every 30 min throughout each day of the experiment, the wind velocity was observed using high-precision anemoscopes with a USB interface and a Uni-T digital wind speed anemometer. The gadget records instantaneous wind speed, ambient temperature, altitude, and atmospheric pressure. The average wind speeds were calculated for the experimental days.

To avoid condensate re-evaporation, the still’s hourly condensate production was collected in a bottle using a graduated jar cylinder. The experimental data was analyzed by evaluating the accuracy of the observed parameters, including temperatures, solar radiation, wind speed, and total dissolved solids (TDS). The level of uncertainty in the experimental results was calculated by determining the minimum mistake using the ratio of the least count to the minimum value. All values were found to be small compared to the obtained data and within the allowable range of the measurements as shown in Table 1.

**Table 1:** Uncertainties in the measured and calculated parameters

Instrument	Unit	Accuracy	Range	Error
Solar power meter	W/m^2^	± 1.0	0–2000	1.5%
K-type thermocouples	°C	± 0.1	− 100–200	0.3%
Productivity	ml	± 2.0	0–500	2.0%
The wind speed	m/s	± 0.1	0–30	2.5%
TDS	ppm	± 1.0	0–1999	2.2%

The experiments were conducted for a period of 4 months, from June to September 2022, between 6:00 AM and 6:00 PM. The temperatures inside the solar still were recorded every 60 min. At the end of each working day, the total water production, water desalination productivity, solar radiation, and wind speed were evaluated. Both solar stills were tested using the same water depths and all measurements were recorded and documented during each experiment.

## The thermal efficiency study

The calculated thermal efficiency of the conventional solar still is determined as reported by the formula of Kabeel and Abdelgaied (2016) and Suraparaju and Natarajan (2021):

1 $${\upeta }_{\mathrm{th}}= \frac{{\mathrm{m}}_{\mathrm{w}}\times\uplambda }{\mathrm{A}\times \mathrm{I}\times \Delta \mathrm{T}}$$

where A is the area of the absorber basin, m^2^, I is the daily average incident solar energy in W/m^2^, and Δt is the cumulative measurements duration in seconds. ${\mathrm{m}}_{\mathrm{w}}$ is the freshwater productivity in kilograms.

2 $$\mathrm a\mathrm n\mathrm d\;\mathrm\lambda=3.1615\times\left(10^6-761.6\times{\mathrm T}_{\mathrm a}\right),\mathrm{if}\;{\mathrm T}_{\mathrm a}>70$$

3 $$\begin{array}{c}\uplambda =2.4935 \times \left({10}^{6}-947.79\times {\mathrm{T}}_{\mathrm{a}}+0.13132\times {{\mathrm{T}}_{\mathrm{a}}}^{2}- 0.004794\times {{\mathrm{T}}_{\mathrm{a}}}^{3}\right)\\ {\mathrm{T}}_{\mathrm{a}}=\frac{{\mathrm{T}}_{\mathrm{water}}+{\mathrm{T}}_{\mathrm{glass}}}{2}\end{array}$$

## Cost evaluation analysis

The primary objective of solar still design is to produce drinkable water in remote, isolated regions at the lowest possible cost (Omara et al. 2014). To achieve this, the costs of various solar stills were compared. The cost analysis of the desalination unit involves several calculation elements, including the capital recovery factor (CRF), fixed annual cost (FAC), sinking fund factor (SFF), annual salvage value (ASV), average annual productivity (AAP), and annual cost (AC). Additionally, the solar still requires an annual maintenance operational cost (AMC) for frequent water filling, distilled water collection, transparent cover cleaning, and salt buildup removal. As the system ages, maintenance costs increase, and 10% of the net current cost has been allocated for maintenance costs. The price of distilled water per liter (CPL) can be determined by dividing the annual cost of the system (AC) by the annual average productivity (AAP) of the solar still. The calculation parameters mentioned above can be expressed as:

4 $$CRF=i{{(1+}i)}^Z{/\lbrack(1+}i)^Z-{1\rbrack}$$

5 $$\text{FAC }=\text{ PCC }({\text{CRF}})$$

6 $$SFF=i/{\lbrack(1+i)}^Z-{1\rbrack}$$

7 $$\text{S }=0.\text{2 (PCC)}$$

8 $$\text{ASV }=({\text{SFF}})\text{ S}$$

9 $$\text{AMC }=0.\text{15 FAC}$$

10 $$\text{AC }=\text{ FAC }+\text{ AMC}-{\text{ASV}}$$

11 $$\text{CPL }=\text{ AC }/\text{ AAP}$$

## Payback period (np)

The payback period refers to the duration taken by a device to offset the cost of investment. If the payback period is represented by n_p_ and the interest rate by i, then the net cash flow at the end of each year is CF, and it can be expressed using the formula proposed in Tiwari et al. (2017):

12 $${\mathrm{P}}_{\mathrm{s}}=\sum\nolimits_{\mathrm{t}=1}^{\mathrm{t}={\mathrm{n}}_{\mathrm{p}}}\left[{\mathrm{CF}}_{\mathrm{t}}\mathrm{ x }{\left(1+\mathrm{i}\right)}^{-{\mathrm{n}}_{\mathrm{p}}}\right]$$

If each year’s net cash flow (CF_t_) is the same, then:

13 $${\mathrm{P}}_{\mathrm{s}}=\mathrm{CF X }{\mathrm{F}}_{\mathrm{RP},\mathrm{i},\mathrm{n}}$$

where, factor ${\mathrm{F}}_{\mathrm{RP},\mathrm{i},\mathrm{n}}$ is employed to handle the interest rate (IR), which is estimated as shown below.

14 $${\mathrm{F}}_{\mathrm{RP},\mathrm{i},\mathrm{n}}= \frac{{[1+\mathrm{i}]}^{{\mathrm{n}}_{\mathrm{p}}}-1}{\mathrm{i X }{[1+\mathrm{i}]}^{{\mathrm{n}}_{\mathrm{p}}}}$$

With simplifying Eq. 14, the payback time is:

15 $${\mathrm{n}}_{\mathrm{p}}= \frac{\mathrm{ln}\left[\frac{\mathrm{CF}}{\mathrm{CF}-\left({\mathrm{P}}_{\mathrm{s}}\mathrm{ X i}\right)}\right]}{\mathrm{ln}\left[1+\mathrm{i}\right]}$$

## Energy matrices

The evaluation of any renewable technology relies on several key parameters, including the life cycle conversion efficiency (LCCE), the energy production factor (EPF), and the energy payback time (EPBT). These parameters can be calculated as follows:

### Energy payback time (EPBT)

According to the energy and exergy approach used in this study, EPBT can be calculated as in Dincer (2002) and PRAKASH and BANSAL (1995):

16 $${(\mathrm{EPBT})}_{\mathrm{en}}=\frac{\mathrm{Embodied}\;\mathrm{energy}\;\mathrm{in}\;({\mathrm E}_{\mathrm{in}})}{\mathrm{Annual}\;\mathrm{energy}\;\mathrm{out}\;({\mathrm E}_{\mathrm{out},\mathrm{ann}})}$$

17 $${(\mathrm{EPBT})}_{\mathrm{ex}}=\frac{\mathrm{Embodied}\;\mathrm{energy}\;\mathrm{in}\;({\mathrm E}_{\mathrm{in}})}{\mathrm{Annual}\;\mathrm{energy}\;\mathrm{out}\;({\mathrm E}_{\mathrm{ex},\mathrm{ann}})}$$

where ${\mathrm{E}}_{\mathrm{out},\mathrm{ ann}}$ is the solar stills annual useful energy (kWh).

### Energy production factor (EPF)

The energy production factor EPF can be determined as in Singh et al. (2016) and Tiwari and Mishra (2012):

18 $${(\mathrm{EPF})}_{\mathrm{en}}=\left[\frac{\mathrm{Embodied}\;\mathrm{energy}\;\mathrm{in}\;({\mathrm E}_{\mathrm{in}})}{\mathrm{Annual}\;\mathrm{energy}\;\mathrm{out}\;({\mathrm E}_{\mathrm{out},\mathrm{ann}})}\right]^{-1}$$

19 $${(\mathrm{EPF})}_{\mathrm{ex}}=\left[\frac{\mathrm{Embodied}\;\mathrm{energy}\;\mathrm{in}\;({\mathrm E}_{\mathrm{in}})}{\mathrm{Annual}\;\mathrm{energy}\;\mathrm{out}\;({\mathrm E}_{\mathrm{ex},\mathrm{ann}})}\right]^{-1}$$

### Life cycle conversion efficiency (LCCE)

LCCE of any enhanced solar stills can be determined as (Tiwari and Mishra 2012), (Sahota et al. 2017):

20 $${(\mathrm{LCCE})}_{\mathrm{en}}= \frac{\left( {\mathrm{E}}_{\mathrm{out},\mathrm{ann}}\mathrm{ X n}\right) - ( {\mathrm{E}}_{\mathrm{in}})}{ {\mathrm{E}}_{\mathrm{sol}\left(\mathrm{ en}\right),\mathrm{ann}}\mathrm{ X n}}$$

21 $${(\mathrm{LCCE})}_{\mathrm{ex}}= \frac{\left( {\mathrm{E}}_{\mathrm{ex},\mathrm{ann}}\mathrm{ X n}\right) - ( {\mathrm{E}}_{\mathrm{in}})}{ {\mathrm{E}}_{\mathrm{sol}\left(\mathrm{ ex}\right),\mathrm{ann}}\mathrm{ X n}}$$

where, ${\mathrm{E}}_{\mathrm{out},\mathrm{ann}}$ is the yearly output solar energy, i.e., yield production, ${\mathrm{E}}_{\mathrm{in}}$ is the embodied energy, ${\mathrm{E}}_{\mathrm{sol}\left(\mathrm{ en}\right),\mathrm{ann}}$ is the yearly solar energy retrieved or incident on the solar still (total input energy), ${\mathrm{E}}_{\mathrm{ex},\mathrm{ann}}$ is the gain yearly exergy, and *n* is the solar still existence period and the ${\mathrm{E}}_{\mathrm{sol}\left(\mathrm{ ex}\right),\mathrm{ann}}$ is the input yearly solar exergy.

### Exergo-economic analysis

Exergo-economic analysis is an economic evaluation technique that uses exergy analysis. Its aim is to establish a methodology for cost-effective system performance improvement, optimal design, and redesign. The exergoeconomic parameters of the enhanced solar stills can be computed as detailed in [42]:

22 $${\mathrm{R}}_{\mathrm{ex}}= \frac{{\mathrm{E}}_{\mathrm{ex},\mathrm{ann}}}{\mathrm{UAC}}$$

### Enviro-economic analysis

The environmental and economic analysis aims to determine whether the use of solar energy or other renewable sources in engineering can reduce carbon dioxide emissions, which are harmful to the environment. Coal energy production in power plants results in an average release of 0.96 kg/kWh of CO_2_ into the atmosphere. However, when taking into account the 20% loss from inefficient household appliances and the 40% loss from transmission and distribution losses, the estimated CO_2_ per kWh value increases to 2.0 kg/kWh [43]. Therefore, the annual CO_2_ mitigation (φCO_2_) from improved solar stills can be calculated using the formula in Sahota and Tiwari (2017a):

On the basis of energy:

23 $${\mathrm{\varphi }}_{\mathrm{CO}2}= \frac{\left({\Psi }_{\mathrm{CO}2}/0.38\right) X{\mathrm{ E}}_{\mathrm{out},\mathrm{ann}} }{{10}^{3}}$$

On the basis of exergy:

24 $${\mathrm{\varphi }}_{\mathrm{CO}2}= \frac{\left({\Psi }_{\mathrm{CO}2}/0.38\right) X{\mathrm{ E}}_{\mathrm{ex},\mathrm{ann}} }{{10}^{3}}$$

where, ${\mathrm{\varphi }}_{\mathrm{CO}2}$ is the average CO_2_ equivalent intensity for coal-based energy production (2.04 kg CO_2_/kWh) or the CO_2_ emissions per the electricity unit. During the useful life of the solar desalination system, an enviroeconomic technique predicts the CO_2_ mitigation industry in terms yearly revenue. As a result, the redesigned solar stills’ environmental cost can be expressed as follows (Sahota and Tiwari 2017b):

25 $${Z}_{\mathrm{CO}2}= {\mathrm{\varphi }}_{\mathrm{CO}2} X {z}_{\mathrm{CO}2}$$

where, $${z}_{\mathrm{CO}2}$$ is the estimated global carbon price of US $10.76 per ton of CO_2_.

## Results and discussion

### Solar still performance

A series of experiments were conducted using seawater with a salinity of TDS 15,300 ppm in a CSS basin with a surface area of 0.8 square meters. The experiments yielded several results related to the number of luffa fibers used inside the basins to obtain higher yield productivity with TDS 211 ppm compared to the yield productivity of conventional solar stills. After identifying the optimum number of luffa fibers to use, various scenarios were tested using the same number of media inside the basins, which were 15 pieces each of natural luffa fiber (NLF), black luffa fiber (BLF), fine steel wool (FSW), and steel wool pads (SWP). The results indicated that filling the entire surface with natural luffa fiber resulted in a higher surface temperature compared to not using luffa fiber due to heat obstruction inside the fissures of the luffa fiber.

The basin temperature, surface water temperature, space inside the basin temperature, and internal surface of the glass were measured and recorded as in the following figures. A K-type thermocouple was used with a 3 cm water depth inside the basin. The experiments revealed that covering the full basin area with natural luffa fibers had a negative impact on the yield productivity of the single slope solar still.

**Fig. 4:**
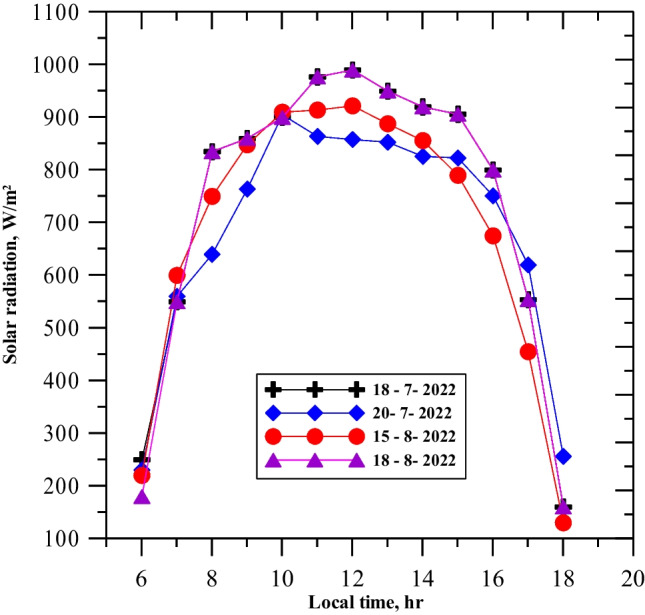
Variation of solar radiation intensity with local time

**Fig. 5:**
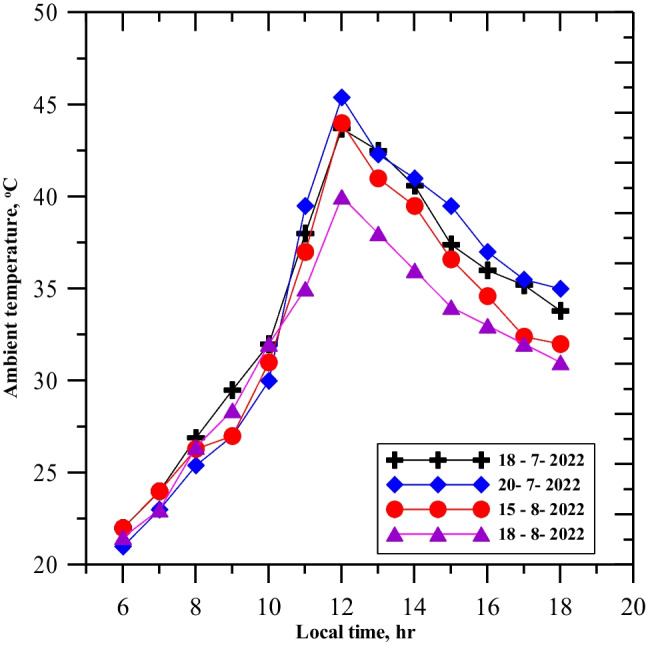
Variation of ambient temperature on the testing days

When natural luffa fiber was used to cover the full basin area, there was a negative impact on the yield productivity of the single slope solar still. However, better performance was achieved when only 40% of the area was covered with 15 pieces of natural luffa fiber, as using a large number of luffa inside the basin decreased productivity due to excessive absorption and little evaporation. The solar radiation and ambient temperature were measured for 4 days (July 18th and 20th, August 15th and 18th, 2022) and recorded in Figs. 4 and 5, respectively. The solar radiation ranged from 400 to 995 W/m^2^, with the highest recorded at noon on July 18th and August 18th, and the lowest recorded at 17:00 on August 15th. The ambient temperature was highest at noon on July 20th (47 °C) and lowest at noon on August 18th (40 °C), with a 10-degree temperature difference between 6:00 AM and 6:00 PM for the same day and an average ambient temperature of about 37 °C. The recorded temperatures were graphically plotted against time from morning to evening for the four testing days.

**Fig. 6:**
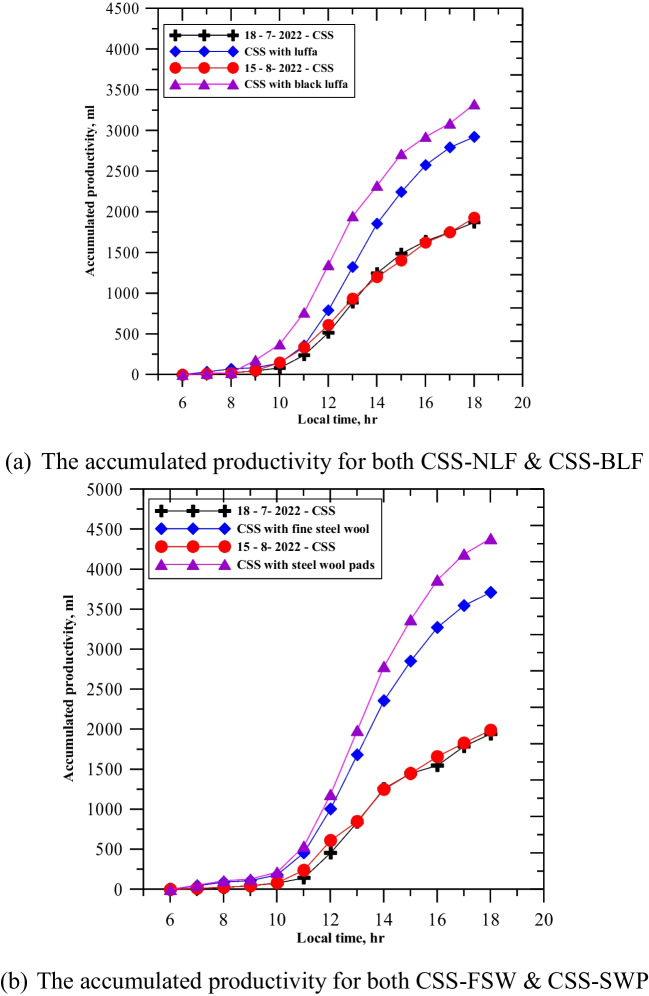
Accumulated productivity of the enhanced solar still compared with the conventional one. **a** The accumulated productivity for both CSS-NLF and CSS-BLF. **b** The accumulated productivity for both CSS-FSW and CSS-SWP

The comparison experiment between traditional CSS and CSS-NLF, CSS-BLF, CSS-FSW, and CSS-SWP was conducted on 2 days, July 18th and August 15th, 2022, from 6:00 AM to 6:00 PM. The productivity measurements were made with reference to the traditional CSS purification system. Figure 6a shows the experimental results of productivity quantity between CSS and natural luffa fiber (NLF) and black luffa fiber (BLF). The results for CSS-NLF were measured on July 18th, and the results for CSS-BLF were measured on August 15th. The cumulative productivity for both CSS and CSS-NLF was plotted, showing that CSS-NLF had higher productivity than CSS on the same day, with an accumulative productivity of 3400 ml, compared to 1900 ml with CSS, representing over 77% increase in productivity. Similarly, CSS-NLF produced 2900 ml compared to 1800 ml for CSS, representing over 60% increase in productivity.

Figure 6b shows a comparison between CSS and two other solar still types: FSW on July 18th and SWP on August 15th. The reference point for comparison was CSS, and the improvements in porosity, capillary rise, absorbency, and heat transfer coefficient of the fibers all contributed to higher water temperatures and consequently higher production for the four cases relative to CSS. As previously mentioned, using 15 pieces of the materials inside the absorption basin was found to be the optimum number for improving accumulative productivity. On July 18th, the accumulated productivity was 3750 ml using CSS-FSW compared to 2000 ml using CSS, resulting in a productivity increase of over 87%. The highest productivity was recorded on August 15th with CSS-SWP, which produced 4400 ml compared to 2010 ml with CSS, resulting in an accumulated productivity increase of 118%.

The productivity of the solar still was improved by incorporating natural and metal fibers into the absorber basin. The addition of metal fibers resulted in an increased evaporation rate, making the process occur more rapidly. The number of pores in the basin, along with the quantity of water absorbed, was easier to evaporate by using metal fibers. On the other hand, the inclusion of natural luffa fibers hindered the penetration of solar rays into the basin, which reduced the heating intensity. However, due to the adsorption properties of the natural luffa fiber, the water quality was better compared to that of FSW and SWP.

**Fig. 7:**
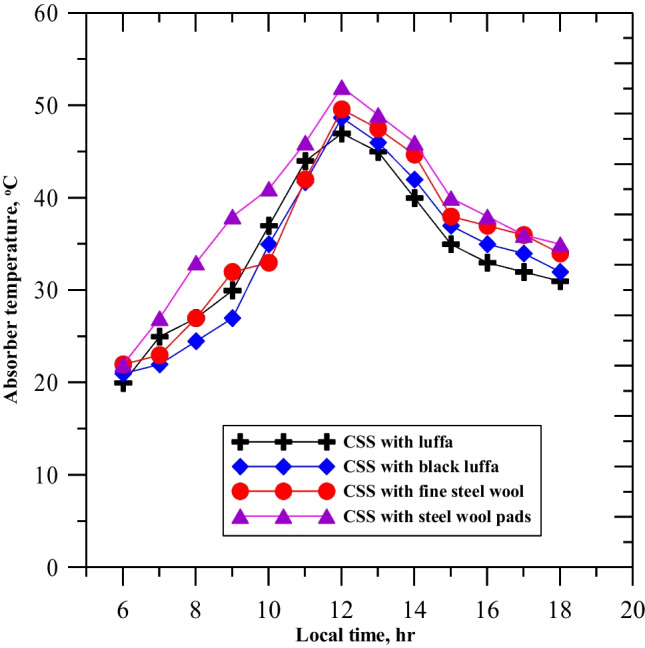
Variation of the absorber temperature for the enhanced solar still on the testing days

Figure 7 shows the absorber basin temperatures of the solar still with natural luffa fiber, black luffa, fine steel wool, and steel wool pads. CSS with steel wool pads had higher temperatures than the other systems, thanks to the inclusion of solar radiation inside the porous steel fibers and natural fibers in the absorber basin. The highest absorber basin temperatures were reported for CSS-SWP at 52 °C, followed by CSS-FSW at 49 °C, CSS-BL at 48 °C, and CSS-NLF at 46 °C.

**Fig. 8:**
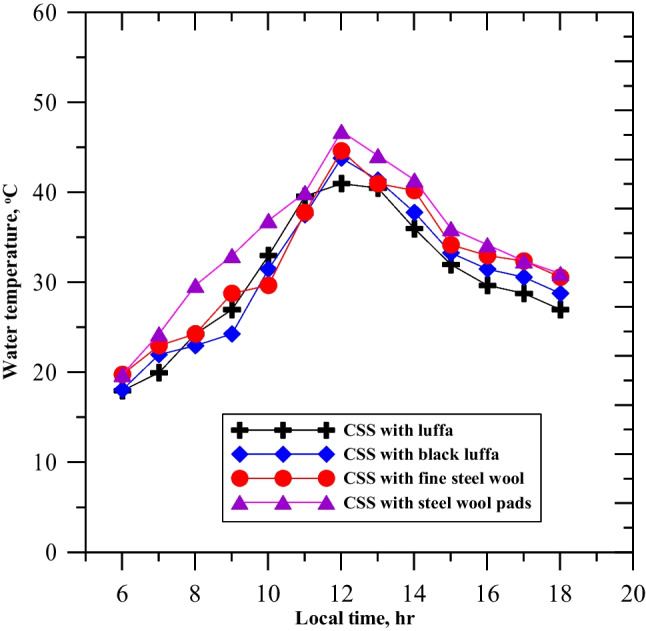
Variation of the water temperature for the enhanced solar still on the testing days

Figure 8 depicts the water temperature in the four cases of the experiments. The water temperature in CSS with natural luffa fiber was slightly higher due to the inclusion of seawater into the fibers but had a low heat-storing capacity, which dissipated the stored heat to the water very rapidly. CSS with steel wool pads had the highest water temperature due to the solar heat stored inside the water and the metal fibers. CSS with black luffa had more heat-storing capacity, leading to an increase in water temperature, whereas fine steel enhanced the energy absorbed and stored in the basin, leading to a higher water temperature than CSS with luffa fibers but less than CSS with steel wool pads. The highest water temperatures reported at noon 12:00 PM were for CSS with steel wool pads at 48 °C, followed by CSS with fine steel wool at 46 °C, CSS with black luffa at 44 °C, and CSS with natural fibers at 41 °C, respectively.

**Fig. 9:**
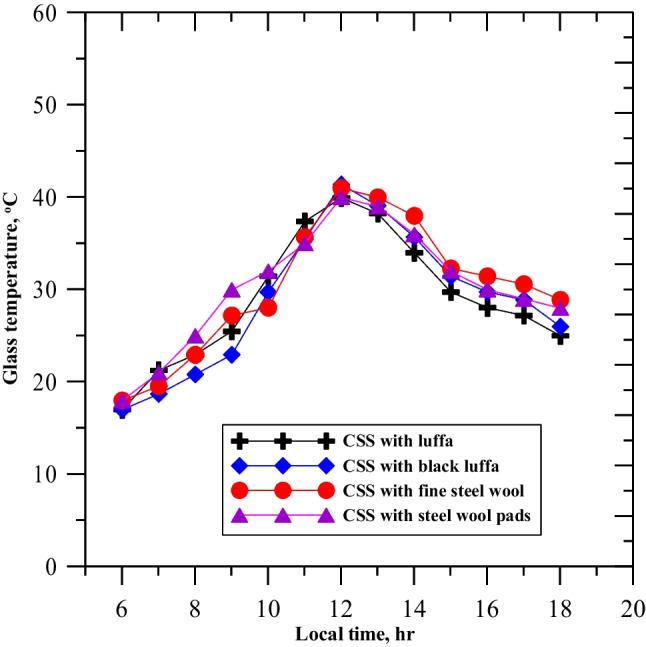
Variation of the glass temperature for the enhanced solar still on the testing days

The study conducted four experiments using a conventional solar still and 15 natural luffa fibers, black luffa fibers, fine steel wool, and steel wool pads. Figure 9 shows the glass surface temperatures for each scenario from 6:00 to 18:00, recorded hourly. The results indicated that the lowest temperature was recorded for the conventional solar still with black Luffa fiber from 6:00 to 10:30 and for CSS with fine steel wool and steel wool pads from 10:30 to 12:00. The highest temperature recorded for the glass surface was 41 °C at noon for all four experiments. However, from 12:00 to 18:00, CSS with fine steel wool recorded the highest temperature. The presence of porous luffa fiber in the basin water resulted in the lowest glass temperature for CSS with luffa fiber due to the minimal occurrence of free convection current. Direct solar radiation on the CSS surface was one of the reasons for glass temperature variations. Table 2 summarizes the experiment’s outcomes, including productivity and efficiency for each of the five set-up systems.

**Table 2:** Comparison between productivity increases according to the conventional one

No	Parameter	CSS-NLF	CSS-BL	CSS-FSW	CSS-SWP
1	Productivity, l/m^2^	2.923	3.325	3.712	4.384
2	%age of productivity increase	35.96	41.95	48.57	54.62
3	Daily average efficiency, %	21.22	24.71	28.60	32.74

According to the 2022 prices, the conventional solar still (CSS) costs approximately US $285, assuming a minimum average daily production of 1.95 l/m^2^ per day at a water depth of 3 cm, operating 340 days per year with a still life of 10 years. Its total productivity during its lifetime is 6630 l. By using black or natural luffa fibers, fine steel wool, or steel wool pads, the total cost is about US $295, US $290, and US $295, respectively. Assuming the stills operate the same number of days per year and have the same still life as the conventional solar still, the minimum average daily productivity for CSS-NLF, CSS-BL, CSS-FSW, and CSS-SWP is assumed to be 2.9, 3.4, 3.75, and 4.4 l/m^2^ per day, respectively. The total productivity during the still life for CSS-NLF, CSS-BL, CSS-FSW, and CSS-SWP is 9860, 11560, 12750, and 14960 l, respectively. Table 3 shows the cost estimation for the different components used in the experimental tests, while Table 4 compares the cost of the conventional solar still with modified solar stills with different modifications. In this study, the interest rate is 12% per year, and the number of life years is estimated to be 10 years. The CSS enhanced with steel wool pads has the lowest cost per liter among the other enhancement methods.

**Table 3:** Cost estimation for the components of the conventional and modified solar stills according to year of 2022

Component	CSS, ($)	CSS-NLF, ($)	CSS-BL, ($)	CSS-FSW, ($)	CSS-SWP, ($)
Galvanized iron sheets	30	30	30	30	30
Glass cover	25	25	25	25	25
Support legs and wood	125	125	125	125	125
Coating and primers	30	30	30	30	30
Rubber and insulations	30	30	30	30	30
Clamps	15	15	15	15	15
Pipes and valves	30	30	30	30	30
Luffa	-	10	-	-	-
Blacked luffa	-	-	11	-	-
Fine steel wool	-	-	-	5	-
Steel wool pads	-	-	-	-	10
∑ Total cost	**285**	**295**	**296**	**290**	**295**

**Table 4:** The cost comparison between the various methods of enhancement of conventional solar still and the conventional one

Still type	PCC $	AAP L/m^2^	CRF	FAC	SFF	S	ASV	AMC	AC	CPL $/L/m^2^
CSS	285	6630	0.177	50.44	0.057	57.0	3.25	7.56	54.76	0.0082
CSS-NLF	295	9860	0.177	52.21	0.057	59.0	3.36	7.83	56.68	0.0058
CSS-BL	296	11,560	0.177	52.39	0.057	59.2	3.37	7.86	56.87	0.0049
CSS-FSW	290	12,750	0.177	51.33	0.057	58.0	3.33	7.70	55.72	0.0044
CSS-SWP	295	14,960	0.177	52.21	0.057	59.0	3.36	7.83	56.68	0.0034

### Payback period (np)

**Fig. 10:**
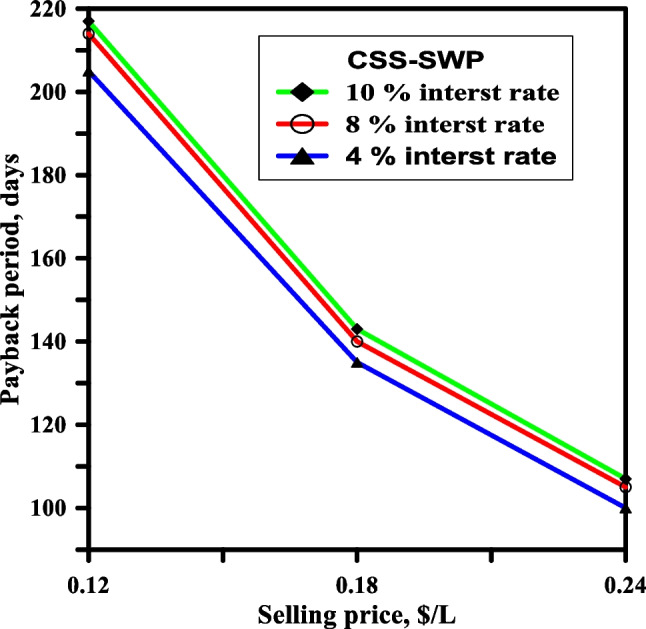
The effect of the sale price and the interest rate on the time needed to pay back the debt for CSS—SWP (life span 10 years and water depth of 3 cm)

The study examined the effect of the selling price of purified distilled water on the payback period of CSS-SWP at a water depth of 3 cm over a period of 10 years with varying interest rates. The results indicated that the minimum payback period was achieved for CSS-SWP due to its high yield. Figure 10 illustrates the impact of selling price and interest rate on the payback period for CSS-SWP. The figure reveals that the payback period decreases as the selling price for distilled water increases, while it increases with an increase in the interest rate at each selling price of distilled water. For instance, the payback period of CSS-SWP was about 103 days at a selling price of 0.24 $/l and 4% IR, which is the lowest value among the other solar stills. Moreover, the other solar stills under study exhibited a similar pattern of behavior with respect to the variation of IR and selling price on the payback period. The highest recorded value for the payback period was achieved for CSS, which was 210 days at 4% IR and a selling price of 0.24 $/l for the distilled water.

### Energy matrices

Table 5 presents estimates of the EPBT, EPF, and LCCE for both conventional and modified solar stills at a water depth of 3 cm, based on annual energy and exergy. The EPBT values for CSS-FSW and CSS-SWP are the lowest among the studied modified solar stills, due to their higher productivity. Specifically, on the basis of energy, the EPBT values for CSS-FSW and CSS-SWP are 0.763 and 0.665 years, respectively, while on the basis of exergy, they are 11.846 and 11.235 years, respectively (the lowest among studied modified solar stills).

**Table 5:** Energy matrices (EPBT, EPF, and LCCE) for CSS, CSS-NLF, CSS-BL, CSS-FSW, and CSS-SWP at water depth of 3 cm on the basis of annual energy and exergy

Basis	CSS			CSS-NLF			CSS-BL		
	EPBT (year)	EPF	LCCE	EPBT (year)	EPF	LCCE	EPBT (year)	EPF	LCCE
Energy	1.868	0.537	− 0.025	1.282	0.780	− 0.020	1.218	0.821	− 0.002
Exergy	13.426	0.074	− 0.2188	12.910	0.077	− 0.1704	12.854	0.078	− 0.1507
Energy	0.763	1.310	0.024				0.665	1.504	0.031
Exergy	11.846	0.084	‒ 0.1039				11.235	0.089	− 0.1011

CSS-FSW and CSS-SWP also have higher EPF values than the other modified solar stills, with values of 1.310 and 1.504, respectively, on the basis of energy, and values of 0.084 and 0.089, respectively, on the basis of exergy. This is due to their higher productivity and energy output, and the margin of embodied for both CSS-FSW and CSS-SWP is minimal. In other words, any decrease in EPBT for the solar still corresponds to an increase in EPT.

**Fig. 11:**
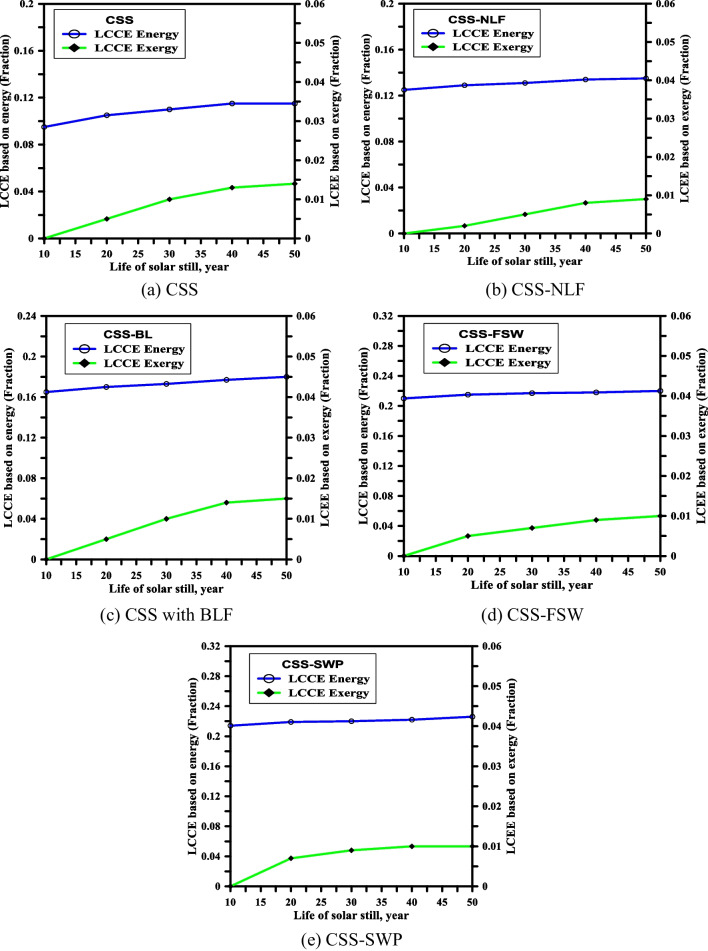
The life cycle conversion efficiency (LCCE) for CSS, CSS-NLF, CSS-BL, CSS-FSW, and CSS-SWP at water depth of 3 cm

Figure 11 shows the variation of LCCE with the existence period (n ranging from 10 to 50 years) for CSS, CSS-NLF, CSS-BL, CSS-FSW, and CSS-SWP, respectively. The values of LCCE for CSS-FSW and CSS-SWP are greater than the corresponding values for the other modified solar stills. Additionally, it can be observed that the values of LCCE for CSS, CSS-NLF, and CSS-BL have negative values for the first year and gradually increase with an increase in the number of lifespan years until they become almost constant from *n* = 40 to *n* = 50 years, as shown in Fig. 11a–c. According to Fig. 11, the LCCE values for all investigated solar stills increase as the systems’ lifespans increase at each of the mentioned water depths. The maximum recorded values of LCCE for CSS-SWP are 0.031 and − 0.1011 on the basis of energy and exergy, respectively.

### Exergo-economic analysis

This section presents an exergo-economic analysis of various solar stills at a water depth of 3 cm, with a focus on exergo-economic parameters (Rex) and their correlation with the unit annualized cost (UAC) at different interest rates (i.e., 4%, 8%, and 10%) over different lifespans (i.e. 30, 40, and 50 years). As expected, the Rex decreases with an increase in interest rate for a fixed duration of the solar stills. Among the modified solar stills studied, the CSS-SWP exhibits the lowest Rex of about 0.0278 kWh/$ at a 30-year lifespan and 10% interest rate.

Exergy analysis plays a vital role in the development and economic analysis of a system since it accounts for the losses due to irreversibilities and waste streams between the input and output energy with respect to the work value. The exergy efficiency of a system depends on the ambient conditions, such as temperature and pressure. Thus, exergy analysis is essential for estimating the process economics, resource utilization, and environmental impacts of a system.

Figure 11a depicts the changes in the levelized cost of energy (LCCE) for CSS, showing that the system’s exergy performance is better at higher lifespans based on energy and exergy analysis. Figure 11b shows the variation in energy and exergy for CSS-NLF, where the energy output is higher than CSS, but the exergy efficiency is about 55% better than that of CSS. Figure 11c shows the exergy efficiency of CSS-BLF to be 75% higher than that of CSS, while Fig. 11d shows a similar exergy efficiency for SWP. Figure 11e shows that the exergy efficiency for FSW is similar to that of CSS-NLF.

For the solar stills with steel wool pads at a water depth of 3 cm, the exergy-based exergo-economic parameter has been determined for lifespans of 30, 40, and 50 years. It has been observed that as the interest rates increase, the exergo-economic parameter (Rex) for fixed solar still lifespans gradually decrease. Similarly, an exergo-economic analysis of a solar still with SWP operating at a water depth of 3 cm can be carried out.

### Enviro-economic analysis

Table 6 presents the environmental benefits of the studied solar stills in terms of CO_2_ reduction and carbon credits. The amount of CO_2_ mitigated annually (φCO_2_) and the corresponding carbon credits are evaluated for solar stills operating at a water depth of 3 cm. The analysis reveals that the amount of energy and exergy-based CO_2_ mitigation per year increases with decreasing water depth, due to the higher annual improvements in production and exergy at shallow water depth. Moreover, the amount of CO_2_ mitigated generally increases with increasing solar still productivity, with the energy-based CO_2_ mitigation being greater than the exergy-based mitigation. Among the studied solar stills, the CSS-SWP shows the highest annual productivity and thus the highest amount of CO_2_ mitigation. Specifically, the energy-based CO_2_ mitigations for CSS, CSS-NLF, CSS-BL, CSS-FSW, and CSS-SWP are 2.22, 4.24, 5.38, 7.67, and 8.77 tons per year, respectively, while the exergy-based mitigations are 0.108, 0.128, 0.135, 0.156, and 0.173 CO_2_ per year, respectively. Additionally, the carbon credits earned by each solar still are presented in Table 6. The carbon credits increase with the increase in CO_2_ mitigation. The annual carbon credits earned by CSS, CSS-NLF, CSS-BL, CSS-FSW, and CSS-SWP are 23.88, 45.62, 57.89, 82.53, and 94.37, respectively, on an energy basis, while the values on an exergy basis are 1.16, 1.38, 1.45, 1.68, and 1.86, respectively.

**Table 6:** Enviroeconomic analysis of the studied solar stills on the basis of annual energy and exergy

Basis	CSS			CSS-NLF		
	Energy production cost ($)	Co_2_ mitigated (ton)	Environmental cost (carbon credit, $)	Energy production cost ($)	Co_2_ mitigated (ton)	Environmental cost (carbon credit, $)
Energy	31.85	2.22	23.88	40.90	4.24	45.62
Exergy	4.43	0.108	1.16	4.02	0.128	1.38
	CSS-BL			CSS-FSW		
	Energy production cost ($)	Co_2_ mitigated (ton)	Environmental cost (carbon credit, $)	Energy production cost ($)	Co_2_ mitigated (ton)	Environmental cost (carbon credit, $)
Energy	48.53	5.38	57.89	50.63	7.67	82.53
Exergy	5.12	0.135	1.45	5.90	0.156	1.68
	CSS-SWP					
	Energy production cost ($)	Co_2_ mitigated (ton)	Environmental cost (carbon credit, $)			
Energy	52.66	8.77	94.37			
Exergy	4.83	0.173	1.86			

## Comparison with previous publications

To validate the performance of the enhanced solar still, the experimental results were compared with previously published measurements from Egypt and other countries, as shown in Table 7 (Dhivagar and Kannan 2022), (Dhivagar et al. 2022b), (Dhivagar 2021). The table demonstrates that the annual productivity of the modified solar still with NLF, BL, FSW, and SWP was approximately 9860, 11560, 12750, and 14960 l/m^2^, respectively. The comparison indicates that the modified solar still with SWP yielded more than other solar stills, including the double slope solar still, tilted solar still with wick, and conventional solar still with PV reflector. The increased surface temperature of the CSS-SWP led to a rise in the evaporation rate from the porous-textured metal structure, resulting in the highest productivity of the CSS-SWP and CSS-FSW systems. The absorber’s larger surface area, made possible by its fibers, prevented more incoming radiation from reaching the water and significantly lowered the water’s temperature rise. It was observed that the optimal number of fibers in the absorber basin improved the evaporation rate more effectively compared to a higher number of fibers. In future research, preheating systems could be employed to improve productivity and energy and exergy, depending on latent and sensible heating (Dhivagar 2021).

**Table 7:** The comparison with previous publications

No	Reference	Type of solar still	Annual productivity, l/m^2^	CPL, $/l/m^2^
1	(Dwivedi and Tiwari 2008), (Dwivedi and Tiwari 2008)	Double-slope solar still	464.68	0.01
2	(Sharon et al. 2017), (Sharon et al. 2017)	Tilted solar still with wick	13,600	0.04
3	(Elbar et al. 2019), (Elbar et al. 2019)	Conventional solar still with PV reflector	728.62	0.0155
4	(Sharshir et al. 2020)	Modified basin type double slope multi–wick solar still with black cotton wick	2583.99	0.0074
5	(Dhivagar et al. 2021a)	Using gravel coarse aggregate sensible heat storage assisted	1537	0.0618
6	(Dhivagar et al. 2022b, d)	Solar still system analysis using block magnets (BMSS) and disc magnets (DMSS)	850.5 for BMSS, 761.4 for DMSS	0.0216 for BMSS and 0.0213 for DMSS
7	(Dhivagar et al. 2022a, b, c, d, e)	Black iron oxide magnetic powder to boost solar radiation absorption	1510.00	0.019
8	(Dhivagar et al. 2022a, b, c, d, e)	Ceramic type rectangular and circular magnets in the basin	1150 and 1030 respectively	0.0021 and 0.0022 respectively
9	Current study	CSS	6630	0.0082
		CSS-NLF	9860	0.0058
		CSS-BL	11,560	0.0049
		CSS-FSW	12,750	0.0044
		CSS-SWP	14,960	0.0034

## Conclusions

Few researchers have explored different ways to improve the performance of solar stills, including the use of porous materials for energy storage. Porous materials can increase the water surface area, which enhances the evaporation rate and improves the solar still productivity. In this study, natural and artificial porous absorbing materials, such as luffa fibers (CSS-NLF), black luffa (CSS-BLF), fine steel wool (CSS-FSW), and steel wool pads (CSS-SWP), were utilized to increase the solar still performance. Two similar solar stills were designed, fabricated, and tested at the Faculty of Engineering, Suez Canal University, under the same climate conditions of Egypt. Various parameters were measured to evaluate the solar still performance using natural and artificial porous absorbing materials. The results showed that the still productivity for conventional solar still (CSS), CSS-NLF, CSS-BLF, CSS-FSW, and CSS-SWP was approximately 1.872, 2.923, 3.325, 3.712, and 4.384 l/m^2^, respectively. The thermal efficiency of the considered solar stills was approximately 17.13%, 21.22%, 24.71%, 28.60%, and 32.74%, respectively. Additionally, cost evaluation analysis was conducted, and the cost per liter (CPL) for the considered solar stills was approximately 0.0082, 0.0058, 0.0049, 0.0044, and 0.0034 $/l/m^2^, respectively. Furthermore, payback period, energy payback time, life cycle conversion efficiency, exergo-economic, and enviro-economic analysis were calculated using natural and artificial porous absorbing materials. Based on energy, the produced annual carbon credits earned by CSS, CSS-NLF, CSS-BLF, CSS-FSW, and CSS-SWP were approximately 23.88, 45.62, 57.89, 82.53, and 94.37, respectively. In addition, on the exergy basis, the carbon credits corresponding values were about 1.16, 1.38, 1.45, 1.68, and 1.86, respectively. Finally, the results of this study were compared with different published experimental researches, and the comparison indicated that the current modifications are a promising method for producing potable water.

## Data Availability

Not applicable.
